# Inhibition of Cell-Free Translation and Replication of Tobacco Mosaic Virus RNA by Exogenously Added 5′-Proximal Fragments of the Genomic RNA

**DOI:** 10.3390/v14091962

**Published:** 2022-09-04

**Authors:** Tetsuya Yoshida, Masayuki Ishikawa, Kazuhiro Ishibashi

**Affiliations:** Division of Plant Molecular Regulation Research, Institute of Agrobiological Sciences, NARO, 2-1-2, Kannondai, Tsukuba 305-8602, Japan

**Keywords:** tobacco mosaic virus, RNA virus, RNA replication, translation

## Abstract

Replication proteins of tobacco mosaic virus (TMV), a positive-sense RNA virus, co-translationally bind to a 5′-proximal ~70-nucleotide (nt) region of the genomic RNA, referred to as the nuclease-resistant (NR) region for replication template selection. Therefore, disruption of the interaction between the viral replication proteins and viral genomic RNA is expected to inhibit the replication of TMV. In this study, we demonstrate that the addition of small RNA fragments (18–33 nts in length) derived from different regions within the NR region inhibit the binding of TMV replication proteins to viral RNA and TMV RNA replication in a cell-free system. Intriguingly, some of the small RNA fragments also inhibited the translation of mRNA in a sequence-nonspecific manner. These results highlight the pleiotropic roles of the 5′-proximal region of the TMV genome.

## 1. Introduction

*Tobacco mosaic virus* (TMV) belongs to the genus *Tobamovirus* of the family *Virgaviridae* [1]. TMV has a positive-sense RNA genome that is about 6400 nucleotides (nts) in length and contains at least four open reading frames (ORFs). TMV genomic RNA is 5′-capped and has a tRNA-like structure at the 3′ end. Two replication proteins, 126-kDa protein, which is encoded by the 5′-proximal ORF, and its translational readthrough product 183-kDa protein, play central roles in TMV RNA replication. TMV 126-kDa protein possesses a methyltransferase (Met) domain, an intervening region (IR), and a helicase (Hel) domain, while the 183-kDa protein also contains a polymerase domain [2]. The 5′ untranslated region (UTR) of TMV RNA, referred to as Ω, is about 70 nts in length, and functions as a translational enhancer [3,4,5].

**Figure 1:**
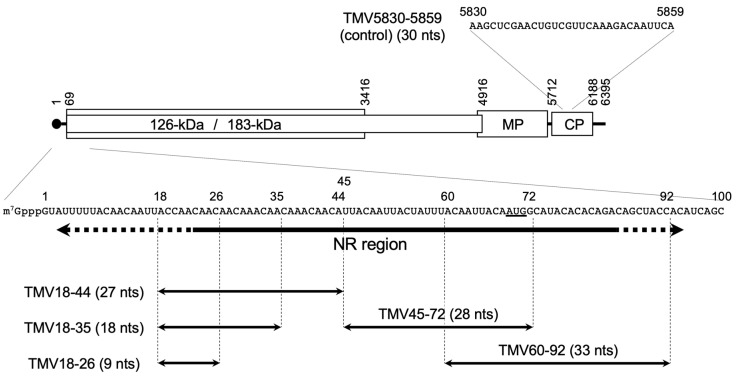
Schematic diagram of the TMV genome structure and small RNA fragments used in this study. The sequence of TMV genome position 1–100 is shown, and the thick arrow roughly indicates the nuclease-resistant (NR) region [8]. Termini of the nuclease-resistant fragments are heterogeneous and indicated by dotted lines. The initiation codon of the ORF for the replication proteins is underlined. Small RNA fragments from the NR region are shown by thin arrows along with numbers representing the positions in the genome. A sequence of the control small RNA fragment from the coat protein (CP) ORF are also shown along with the genome position.

Previously, we established a cell-free translation and replication system for tobamoviral RNA using BYL, an extract of evacuolated tobacco BY-2 protoplasts [6]. Replication of eukaryotic positive-sense RNA viruses occurs on intracellular membranes. In membrane-depleted BYL (mdBYL), which is prepared by removing membranes by centrifugation, tobamoviral RNA is translated to produce replication proteins, and a ribonucleoprotein complex (pre-membrane-targeting complex; PMTC) containing the replication proteins and genomic RNA is formed [7,8]. While the PMTC alone does not show viral RNA replication activity, RNA replication occurs when BYL membranes are supplied post-translationally. In the PMTC, a 5′-proximal ~70-nt region of the viral genome (Figure 1) is bound by TMV replication proteins and protected from nuclease treatment [8]. Here, we refer to this region as the nuclease-resistant (NR) region. PMTC is formed co-translationally, but not post-translationally, which is likely to be a key step in template selection for *cis*-preferential replication of TMV RNA [8,9]. Meanwhile, a C-terminally truncated 126-kDa protein variant called Met-IR, which contains Met and IR but lacks Hel, is able to bind the NR region in TMV RNA post-translationally. This suggests that the Hel domain plays an autoinhibitory role in the full-length 126-kDa protein [8].

Given that the ~70-nt NR region seems too large to be covered by a single protein and that it contains a loosely repetitive sequence, TMV replication proteins may recognize multiple shorter sequence(s) within this region to assemble the PMTC. This study was performed to examine this possibility by investigating whether exogenously added small RNA subfragments of the NR region could competitively inhibit binding of TMV replication protein to the NR region-containing RNA and TMV RNA replication *in vitro*.

## 2. Materials and Methods

### 2.1. RNA Preparation

For affinity purification using StreptoTag, a DNA fragment, 5′-AAGCTTCTAATACGACTCACTATAGGG*GTATTTTTACAACAATTACCAACAACAACAAACAACAAACAACATTACAATTACTATTTACAATTACAATGGCATACACACAGACAGCTACCACATCAGCTTTGCTGGAC*ggatcgcatttggacttctgcccagggtggcaccacggtcggatcc-3′ (the sequence of the T7 promoter is underlined, the sequence of TMV genome position 1–110 is italicized, and the sequence of StreptoTag is shown in lower case) was amplified by PCR using the primers T7-Fw_for_TMV (5′-AAGCTTCTAATACGACTCACTATAG-3′) and TMV110-StreptoTag-Rev (5′-GGATCCGACCGTGGTGCCACCCTGGGCAGAAGTCCAAATGCGATCCGTCCAGCAAAGCTGATGTGGTAGCTGTCTGTGTGT-3′). A capped TMV1-110-st RNA was transcribed from this DNA fragment using the RiboMAX Large Scale RNA Production System—T7 (Promega, Madison, WI, USA) with Ribo m^7^G Cap Analog (Promega).

For production of the TMV Met-IR polypeptide by *in vitro* translation, we constructed a pSPOM [8]-based plasmid, designated as pOMI4-MetIR-FS. pOMI4-MetIR-FS encoded C-terminally FLAG-tagged Met-IR of TMV, in which the sequence of TMV genome position 10–98 was replaced by a sequence with synonymous substitutions in the coding region, 5′-AATATGGCGTATACTCAGACTGCTACGACTTCT-3′ (33 nts) to prevent the binding of Met-IR to the mRNA [8]. A capped RNA was transcribed from *Age*I-linearized pOMI4-MetIR-FS using the RiboMAX Large Scale RNA Production System—SP6 (Promega) with Ribo m^7^G Cap Analog (Promega).

For the *in vitro* translation assay of *Renilla* luciferase mRNA, an *Eco*RI-linearized pMI27 plasmid [10] was used as a template for *in vitro* transcription using the RiboMAX Large Scale RNA Production System—SP6 (Promega) with Ribo m^7^G Cap Analog (Promega, Madison, WI, USA).

TMV-OM RNA was purified from virions by phenol extraction and ethanol precipitation. Small RNA fragments derived from the TMV 5′-proximal region with phosphorothioate linkages and 2′-*O*-methyl modifications were synthesized by Hokkaido System Science (Sapporo, Japan). The sequences and modifications are shown in Table 1.

### 2.2. In Vitro Translation and Replication

Preparation of BYL and mdBYL, and *in vitro* translation and replication reactions, were performed as described previously [11]. TMV-OM RNA at a final concentration of 4 nM, and each small RNA fragment at a final concentration of 0 (control), 0.4, 4, 40, 400, or 4000 nM, were incubated in 25-µL BYL reaction mixtures at 25 °C for 1 h for translation. Aliquots of 20 and 0.3–0.5 µL of the translation mixture were used for replication assay and Western blotting analysis, respectively. *Renilla* luciferase mRNA (final concentration of 0.4 nM) was incubated in an mdBYL reaction mixture with water (control) or each small RNA fragment (final concentration of 4, 40, 400, or 4000 nM) at 25 °C for 1 h.

### 2.3. StreptoTag Affinity Purification

**Figure 2:**
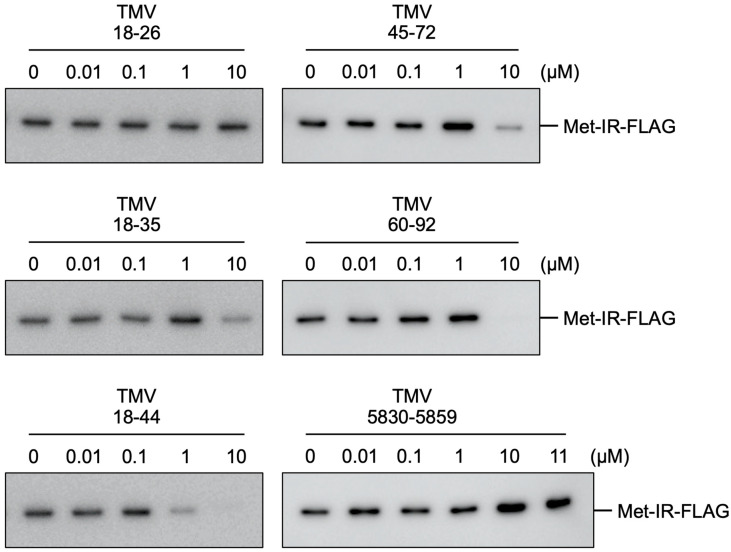
Effects of the addition of small RNA fragments on the binding of FLAG-tagged TMV replication protein fragment (Met-IR) to TMV1-110-st RNA. Met-IR bound to TMV1-110-st-trapped beads, in the presence of the RNA subfragments at the indicated concentrations, was subjected to SDS-PAGE followed by Western blotting using anti-FLAG antibody.

StreptoTag affinity purification was essentially performed as described previously [12,13]. Briefly, 5 μg of yeast tRNA (Thermo Fisher Scientific, Waltham, MA, USA) in 20 µL of column buffer (50 mM HEPES-Na, pH 7.5, 100 mM NaCl, 3 mM MgCl_2_, 0.25 mM DTT) was mixed with 50 µL of dihydrostreptomycin-coupled Sepharose beads (50% slurry) that had been prewashed with column buffer and incubated with rotation for 30 min at 4 °C. Then, 2.1 μg (40 pmol) (Figure 2 and Figure S1A) or 2 µg (37.9 pmol) (Figure S1B) of capped TMV1-110-st RNA (5 µL) was incubated for 3 min at 65 °C and mixed with 5× column buffer (1.25 µL), followed by further incubation for 10 min at 37 °C. The reaction mixture was mixed with dihydrostreptomycin-coupled and yeast tRNA-added Sepharose beads, and rotated for 40 min at room temperature. The beads were washed once with 1 mL of column buffer and twice with 1 mL of TR buffer [11]. Aliquots of 40 μL of mdBYL translation reaction mixture, in which 800 ng of TMV Met-IR-FS RNA had been incubated for 1 h at 25 °C, were mixed with water or 0.4, 4, 40, or 400 pmol of each small RNA fragment (Figure 2 and Figure S1A; or 379 pmol; Figure S1B) for 10 min at 25 °C. These mixtures of Met-IR-FS and small RNA fragments were applied to TMV1-110-st-trapped beads and rotated for 30 min at room temperature. The mixtures were then cooled on ice and 1.6 µL of heparin solution (50 g/L) was added, and the mixtures were then rotated for 20 min at 4 °C. After washing three times with 1 mL of column buffer, protein–RNA complexes were eluted with 60 µL of column buffer containing 15 µM streptomycin. Aliquots of 0.125 (Figure S1A) or 0.5 µL (Figure S1B) of input samples (and flow-through fractions in Figure S1B), and 1.8 (Figure S1A), 3.6 (Figure 2), or 7.2 µL (Figure S1B) of eluates were subjected to Western blotting.

### 2.4. Protein and RNA Analyses

NuPAGE 4–12% Bis-Tris protein gel (Thermo Fisher Scientific, Waltham, MA, USA) was used for SDS-PAGE and Western blotting analyses. The chemiluminescence signals were detected using the LAS-3000 device (FUJIFILM, Tokyo, Japan). For detection of FLAG-tagged Met-IR and untagged full-length replication proteins of TMV, anti-DYKDDDDK antibody (clone 1E6; FUJIFILM Wako Pure Chemical, Osaka, Japan) and antisera against tomato mosaic virus replication proteins [8] were used, respectively. Luciferase activity was measured using the *Renilla* Luciferase Assay System (Promega) and the TD-20/20 luminometer (Promega). Radiolabeled RNA products were separated by 8 M urea–2.4% PAGE and detected using the Typhoon FLA 7000 scanner (GE Healthcare, Chicago, IL, USA). Band intensity of viral genomic RNA was quantified using ImageJ software (National Institutes of Health, Bethesda, MD, USA).

## 3. Results

### 3.1. Inhibition of Binding of Met-IR to the NR Region by RNA Subfragments of This Region

To investigate the function of the NR region, we subdivided the NR region and prepared chemically-synthesized small RNA fragments (9–33 nts) corresponding to TMV genome positions 18–26, 18–35, 18–44, 45–72, and 60–92 (TMV18-26, TMV18-35, TMV18-44, TMV45-72, and TMV60-92, respectively). TMV18-26, TMV18-35, and TMV18-44 were designed to contain different lengths of the AC-rich repetitive sequence, and TMV45-72 and TMV60-92 cover the rest of the NR region (Figure 1). We examined if they competed for the binding of Met-IR to the NR region. An RNA fragment corresponding to TMV genome position 5830–5859 within the ORF encoding coat protein (TMV5830-5859) (Figure 1) was used as a negative control. These small RNA fragments were not 5′-capped but 2′-*O*-methylated at the 3′ termini and partially phosphorothioated (see Materials and Methods and Table 1 for details) for stability in BYL.

The binding of Met-IR to the NR region was examined using StreptoTag, an RNA aptamer that binds to streptomycin [12]. A 5′-capped RNA fragment designated as TMV1-110-st, consisting of the sequence from TMV genome position 1–110 with StreptoTag at the 3′ end, was immobilized on dihydrostreptomycin-conjugated beads. The Met-IR fragment was synthesized by translation in mdBYL, mixed with 0.01, 0.1, 1, or 10 µM of each small RNA fragment, and added to the TMV1-110-st-immobilized beads. TMV5830-5859 (30 nts) was also added at 11 µM as a control to 10 µM of TMV60-92 (33 nts) to adjust the amount of the RNA fragment. After incubation and washing, the trapped TMV1-110-st–Met-IR complex was eluted from the beads in a streptomycin-containing buffer, and Met-IR was detected by Western blotting. TMV5830-5859 (negative control) or TMV18-26 did not inhibit Met-IR binding to TMV1-110-st even at 10 µM or 11 µM (Figure 2). TMV18-35, TMV45-72, or TMV60-92 inhibited binding at 10 µM but not at 1 µM or less (Figure 2). TMV18-44 inhibited binding at 10 or 1 µM, but not at 0.1 or 0.01 µM (Figure 2). Although Met-IR was weakly detected when 10 µM of TMV18-35 and TMV45-72 was added in Figure 2, the Met-IR signal was almost below the limit of detection in other experiments under similar conditions (Figure S1). These results show that TMV18-35, TMV18-44, TMV45-72, and TMV60-92, but not TMV18-26, can compete with TMV1-110-st for Met-IR binding, among which TMV18-44 has the highest activity. In Figure 2, larger amounts of Met-IR were detected in several lanes than in the absence of small RNA fragments (e.g., 1 µM of TMV18-35, 1 µM of TMV45-72, or 10 µM of TMV5830-5859). For TMV45-72 and TMV5830-5859, the results were reproduced in Figure S1A. However, enhanced copurification of Met-IR was not observed in other independent experiments (for TMV5830-5859, see Figure S1B). Thus, the significance of the observed enhancement requires further examination. 

### 3.2. Inhibition of Cell-Free TMV RNA Replication by RNA Subfragments of the NR Region

**Figure 3:**
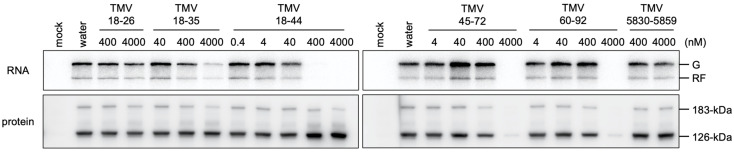
Effects of the addition of small RNA fragments on cell-free translation and replication of TMV RNA. *In vitro* TMV RNA translation–replication assay was performed in the presence of small RNA fragments at various concentrations. *De novo*-synthesized TMV RNA was analyzed by urea-PAGE and autoradiography (upper panel). The accumulation of TMV replication proteins after translation reaction was examined by SDS-PAGE and Western blotting using antisera against replication proteins (lower panel). Positions of the genomic (G) and replicative form (RF) RNAs and 126-kDa and 183-kDa replication proteins are shown on the right.

As the TMV replication protein–genomic RNA interaction is crucial for its RNA replication [8], we investigated whether small RNA fragments from the NR region can inhibit TMV RNA replication *in vitro*. TMV virion RNA (4 nM) was translated in the presence of small RNA fragments (0.4–4000 nM) in BYL, and *de novo* synthesis of TMV-related RNA was detected by incorporation of [α-^32^P]CTP. When TMV18-35, TMV18-44, TMV45-72, or TMV60-92 RNA was added at a concentration of 4,000 nM, the synthesis of TMV-related RNA was inhibited, while TMV5830-5859 or TMV18-26 had a much smaller effect (if any) than other RNA fragments (Figure 3, Figures S2 and S3). These results showed that the small RNA fragments that inhibited the binding of TMV replication protein fragment to the NR region-containing RNA (TMV18-35, TMV18-44, TMV45-72, and TMV60-92) also inhibited the replication of TMV RNA. TMV18-44 inhibited TMV RNA replication at lower concentrations than other RNA fragments in four independent experiments (Figure 3, Figures S2 and S3), suggesting that TMV18-44 has greater inhibitory activity for both processes than the other small RNA fragments. After translation of TMV RNA in the presence of TMV18-26, TMV18-35, TMV18-44, and TMV5830-5859, the levels of TMV replication protein accumulation were comparable to those in the water control (Figure 3 and Figure S2). Therefore, TMV18-35 and TMV18-44 inhibited TMV RNA replication by inhibiting the function of replication proteins, most likely their binding to the NR region, as expected. Unexpectedly, in the presence of higher amounts of TMV45-72 or TMV60-92, the accumulation of TMV replication proteins decreased (Figure 3 and Figure S2). This suggests that these RNA fragments impaired TMV RNA translation. Note that TMV RNA replication was strongly inhibited even in the condition that translation was not obviously inhibited by TMV45-72 or TMV60-92 (Figure S2). This suggests that these fragments have dual functions: inhibition of TMV RNA translation and the binding of replication proteins to viral RNA.

### 3.3. Inhibition of Translation of Nonviral mRNA by RNA Subfragments of the NR Region

**Figure 4:**
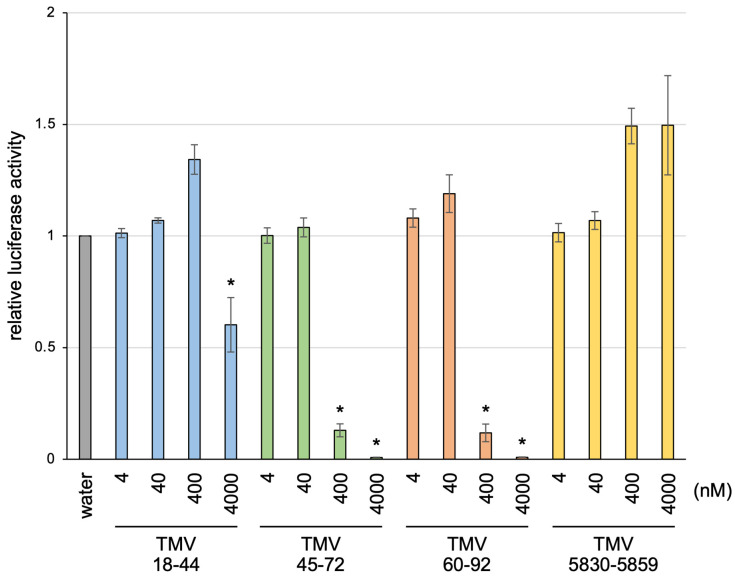
Effects of the addition of small RNA fragments on luciferase mRNA translation. *Renilla* luciferase mRNA was incubated in mdBYL in the presence of water or small RNA fragments at the indicated concentrations, and luciferase activity was measured. The mean and standard error of at least three independent experiments are shown. Values were normalized relative to the water control. Asterisks indicate significant differences by Dunnett’s test compared with TMV5830-5859-added samples in the same molar concentrations (* *p* < 0.05).

We then examined whether translational inhibition by TMV45-72 and TMV60-92 is specific to TMV RNA. *Renilla* luciferase mRNA (0.4 nM) was translated in mdBYL in the presence of TMV18-44, TMV45-72, TMV60-92, or TMV5830-5859, and luciferase activity was measured. In the presence of TMV45-72 or TMV60-92 at higher concentrations, luciferase activity was markedly decreased (Figure 4). In contrast, TMV5830-5859 and TMV18-44 showed no translational inhibitory activity, and much weaker translational inhibitory activity than TMV45-72 and TMV60-92, respectively. This result suggests that TMV45-72 and TMV60-92 inhibit mRNA translation in a sequence-nonspecific manner. 

## 4. Discussion

In this study, we identified 18–33-nt RNAs that inhibited not only the binding of Met-IR to the entire NR region-containing RNA (TMV1-110-st), but also TMV RNA replication when added exogenously to a cell-free translation–replication system. This result suggests that template selection by the replication proteins, despite its *cis*-preferential nature, can be inhibited by small RNAs supplied in *trans*, and therefore that the process is a possible target of anti-tobamoviral treatment.

TMV18-35, TMV18-44, TMV45-72, and TMV60-92, but not TMV18-26, inhibited the binding of TMV Met-IR to TMV1-110-st RNA (Figure 2). It would be possible that these small RNA fragments and TMV1-110-st competitively bind Met-IR. Template selection by TMV replication proteins is *cis*-preferential [9]. During the translation of TMV RNA, nascent polypeptide containing the Met-IR fragment would bind the NR region, thus facilitating *cis*-preferential PMTC formation. The small inhibitory RNAs may be able to access the nascent Met-IR polypeptide before PMTC formation when supplied at high concentrations (400–4000 nM).

Given that TMV18-44 and TMV45-72 do not overlap, the NR region would have multiple Met-IR binding sites. TMV18-44 inhibited the binding of Met-IR to the NR region at lower concentrations than other fragments. Therefore, TMV18-44 is likely to have the highest affinity to TMV replication proteins among the small RNA fragments examined in this study. From these results, we propose a model of PMTC formation in which one or a few molecules of TMV replication protein co-translationally bind to the TMV18-44-containing region first, and then several molecules of replication proteins are assembled to cover the whole NR region. Further studies of the NR region will yield more information about the PMTC formation process.

TMV18-26 RNA is only 9 nts in length and showed a much weaker inhibitory effect on the binding of TMV Met-IR to the NR region-containing RNA and RNA replication than TMV18-35 (18 nts) and TMV18-44 (27 nts). Considering the repetitive nature of the sequences of these RNAs (Figure 1), TMV replication proteins may recognize RNA stretches longer than 9 nts.

TMV45-72 and TMV60-92 inhibited RNA translation (Figure 3 and Figure 4). The translational inhibitory activity of this region may play a role in the viral infection process, such as encapsidation of the genomic RNA at a later stage of infection. Alternatively, the observed translational inhibition may be a consequence of competitive binding to translation-associated factors, because these small RNA fragments contain parts of the TMV 5′ UTR sequence (called Ω; genome position 1–68), which is a well-known translational enhancer [3,4,5]. The poly(CAA) region in Ω is critical for translational enhancement [14]. This region interacts with the host heat shock protein 101 (HSP101), and tobacco HSP101 enhances translation in an Ω-dependent manner in yeast [15,16]. An Ω-HSP101 complex was proposed to recruit host eIF4F to enhance translation [17]. Meanwhile, Agalarov et al. showed that ribosomal particles can initiate translation at Ω in a cap- and eIF4F-independent manner [18]. Therefore, TMV45-72 and TMV60-92 may directly recruit ribosomes or other translation-related factors, and consequently inhibit RNA translation in *trans*. Although TMV18-44 consists of the CAA-rich sequence, this RNA fragment showed a much weaker inhibitory effect on translation than TMV45-72 and TMV60-92 (Figure 3 and Figure 4). Thus, translational inhibition by TMV45-72 and TMV60-92 may not involve HSP101. Further studies regarding the mechanisms of translational inhibition by these RNA fragments would facilitate the understanding of its significance in the TMV infection process.

## Figures and Tables

**Table 1:** Small RNA fragments used in this study.

Name	Sequence (5′–3′)
TMV18-26	A*C*CAACAA*c
TMV18-35	A*C*CAACAACAACAAACA*a
TMV18-44	A*C*CAACAACAACAAACAACAAACAAC*a
TMV45-72	U*U*ACAAUUACUAUUUACAAUUACAAUG*g
TMV60-92	A*C*AAUUACAAUGGCAUACACACAGACAGCUAC*c
TMV5830-5859	A*A*GCUCGAACUGUCGUUCAAAGACAAUUC*a

The asterisks denote the phosphorothioate linkage. 2′-*O*-methylated nucleotides are shown in lower case.

## Data Availability

The data presented in this study are available from the corresponding author upon request.

## Supplementary Materials

The following supporting information can be downloaded at: https://www.mdpi.com/article/10.3390/v14091962/s1, Figure S1: Inhibition of the binding of TMV Met-IR to TMV1-110-st by small RNA fragments (related to Figure 2), Figure S2: Effects of the addition of small RNA fragments on *in vitro* translation and replication of TMV RNA (related to Figure 3), Figure S3: Relative band intensity of viral replication product (related to Figure 3 and Figure S2).
