# Supplementary material for: Inhibition of Cell-Free Translation and Replication of Tobacco Mosaic Virus RNA by Exogenously Added 5′-Proximal Fragments of the Genomic RNA
Source: Viruses. 2022 Sep 4;14(9):1962. doi: 10.3390/v14091962 (PMC9502800; doi:10.3390/v14091962)
Supplement: Supplementary file 1 [file viruses-14-01962-s001.zip › FigS1-3/FigS1-3.pdf.pdf]

Figure S1

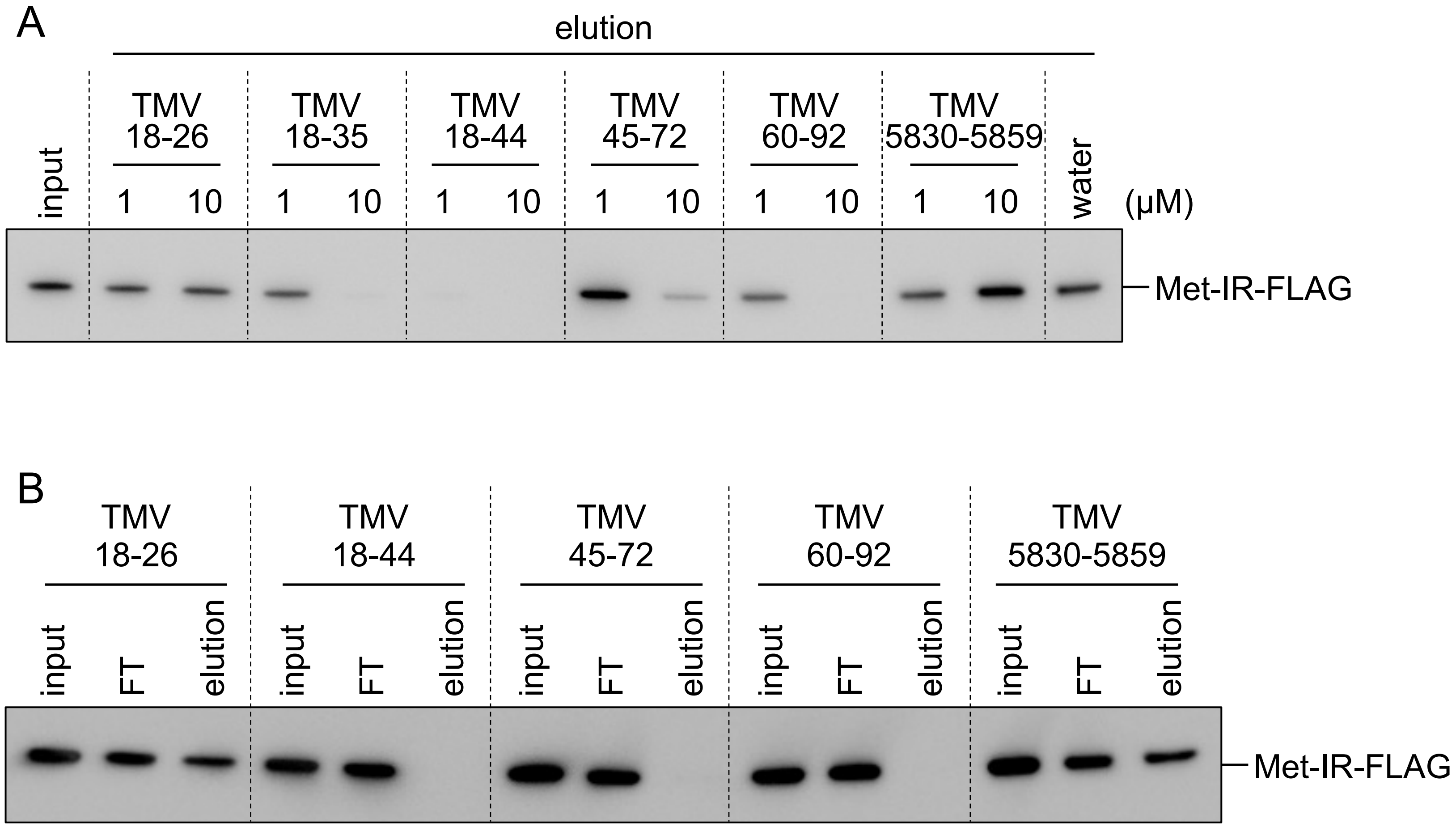

Figure S1. Inhibition of the binding of TMV Met-IR to TMV1-110-st by small RNA fragments (related to Figure 2). The results of independent experiments from Figure 2 using 1 or 10 μM (A) and 9.48 μM (B) of small RNA fragments are shown. The samples before affinity purification (input), from flow-through fractions after affinity purification (FT), and from the eluates from the beads with streptomycin (elution) were analyzed.

Figure S2

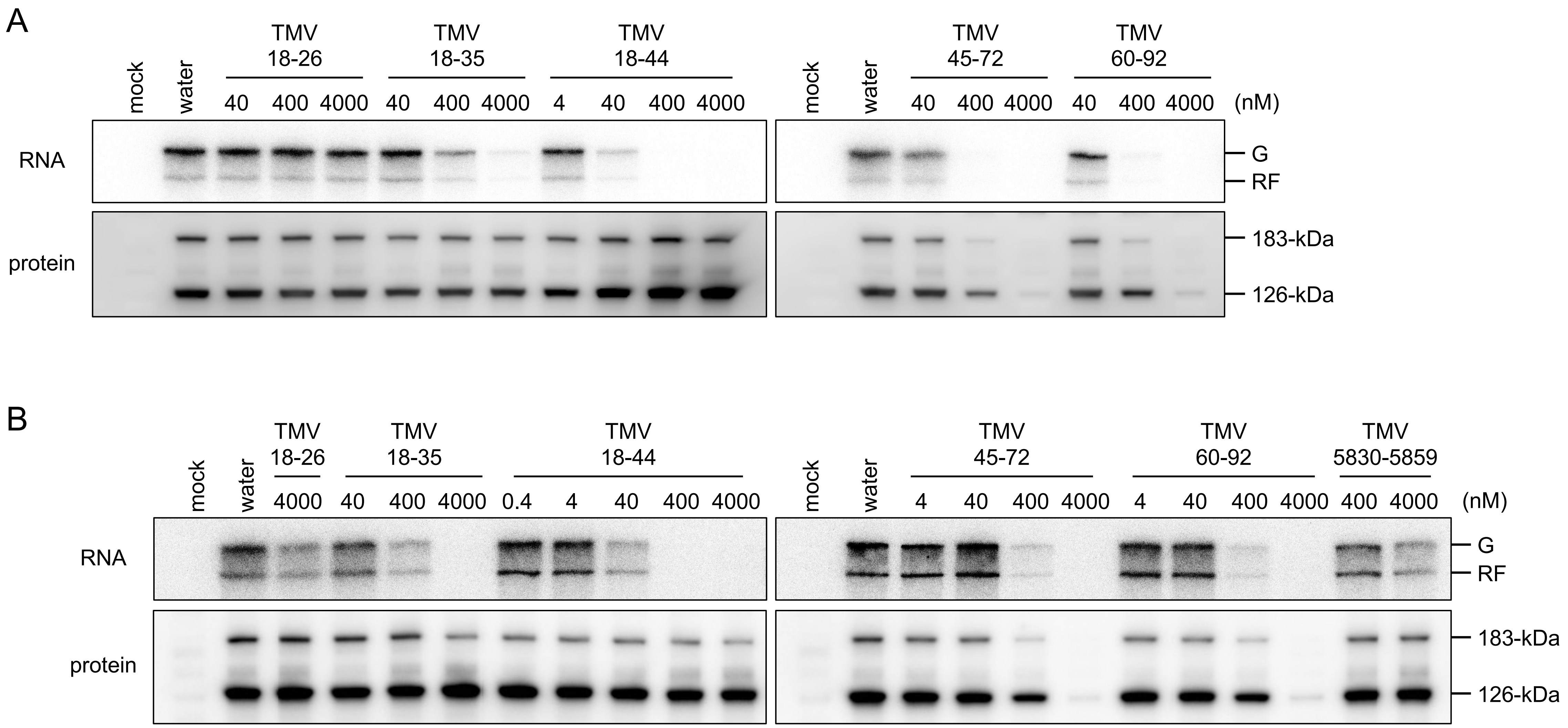
A
TMV 18-26
TMV 18-35
TMV 18-44
mock
water
40 400 4000 40 400 4000 4 40 400 4000
TMV 45-72
TMV 60-92
mock
water
40 400 4000 40 400 4000 (nM)
RNA
protein
G
RF
183-kDa
126-kDa
B
TMV 18-26
TMV 18-35
TMV 18-44
mock
water
4000 40 400 4000 0.4 4 40 400 4000
TMV 45-72
TMV 60-92
TMV 5830-5859
mock
water
4 40 400 4000 4 40 400 4000 400 4000 (nM)
RNA
protein
G
RF
183-kDa
126-kDa

Figure S2

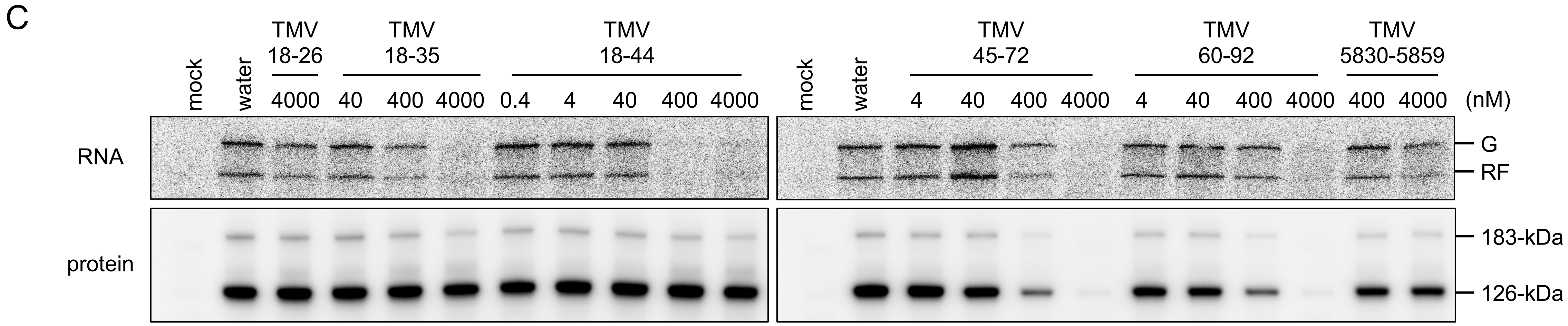

Figure S2. Effects of the addition of small RNA fragments on *in vitro* translation and replication of TMV RNA (related to Figure 3). The results of three additional independent experiments are shown in (A)–(C).

Figure S3

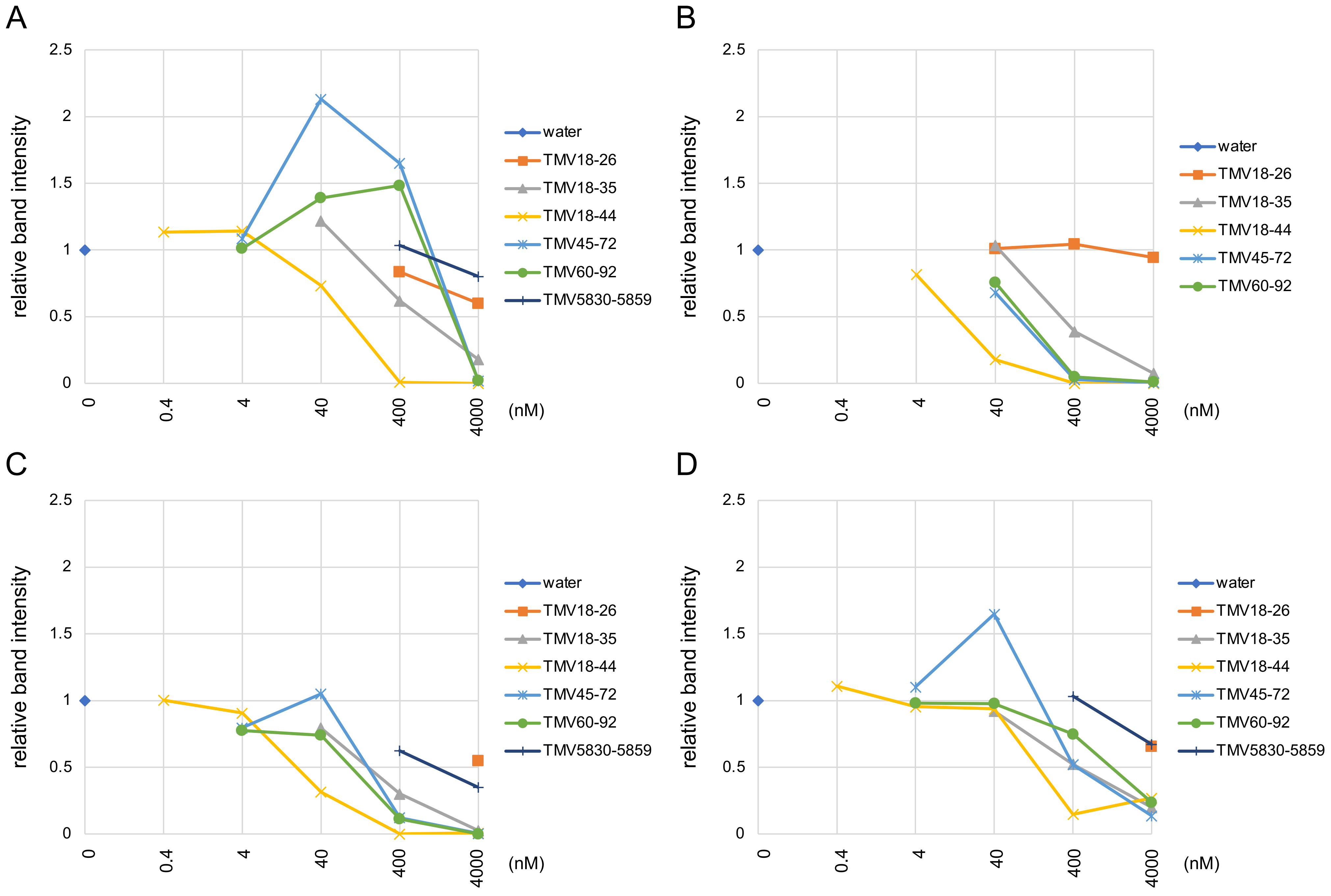

Figure S3. Relative band intensity of viral replication product (related to Figures 3 and S2). Band intensity of *de novo*-synthesized TMV genomic RNA in Figures 3 and S2A–C are shown (A and B–D, respectively). Values were normalized to the water control.
