# Supplementary figures and images for: Inhibition of Cell-Free Translation and Replication of Tobacco Mosaic Virus RNA by Exogenously Added 5′-Proximal Fragments of the Genomic RNA
Source: Viruses. 2022 Sep 4;14(9):1962. doi: 10.3390/v14091962 (PMC9502800; doi:10.3390/v14091962)

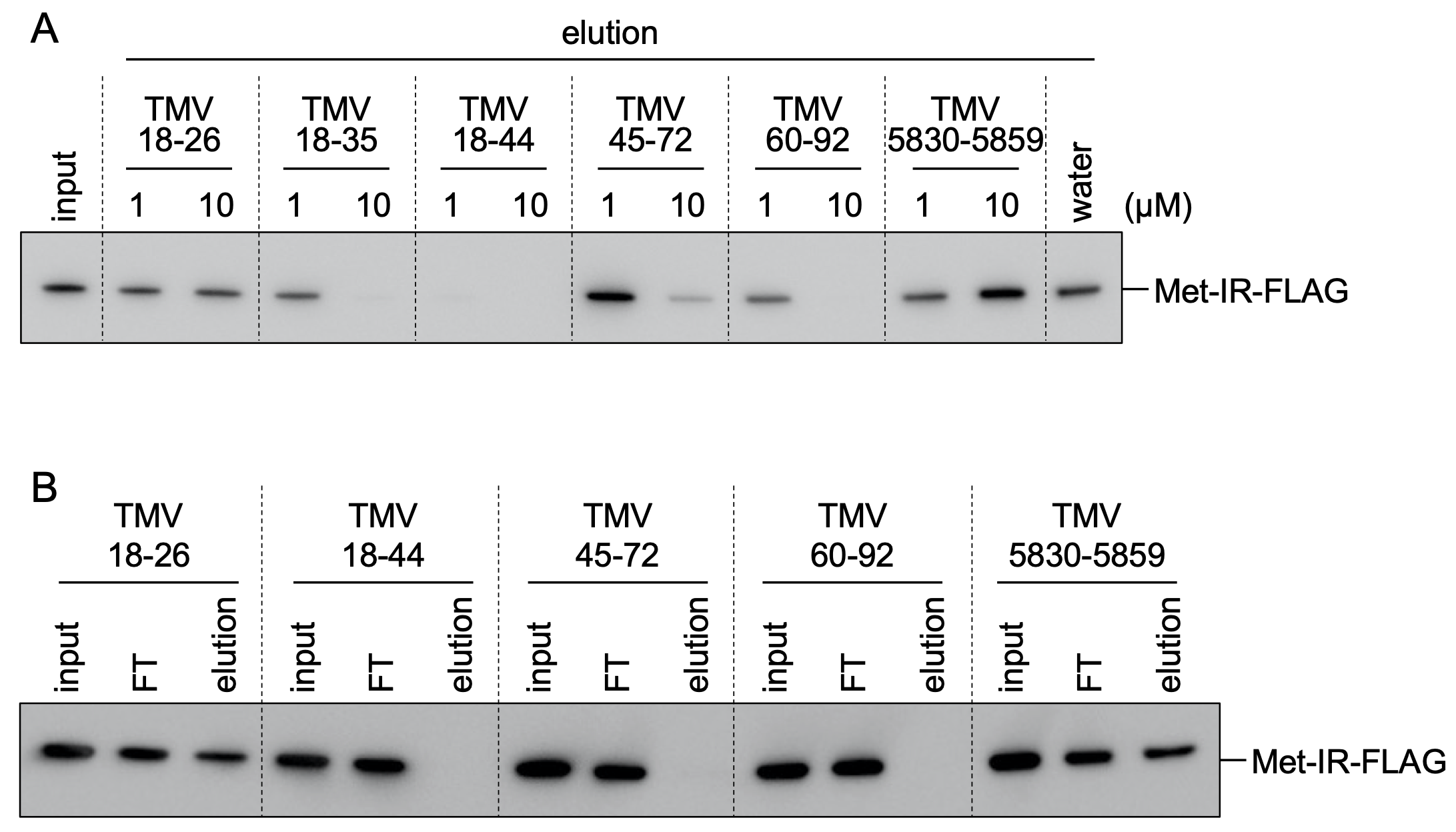

Supplement: Supplementary file 1 [file viruses-14-01962-s001.zip › FigS1-3/FigS1.tiff]

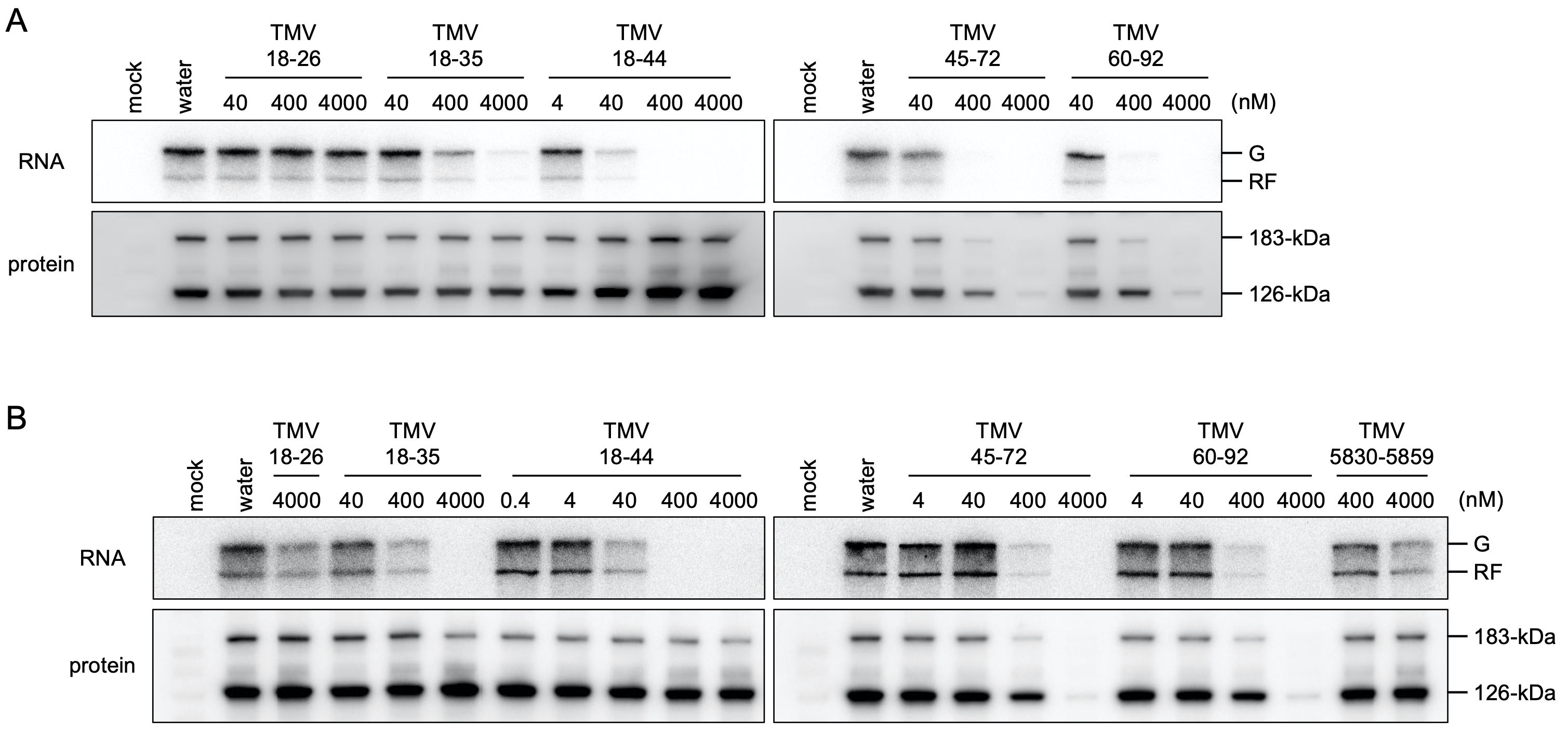

Supplement: Supplementary file 1 [file viruses-14-01962-s001.zip › FigS1-3/FigS2AB.tiff]

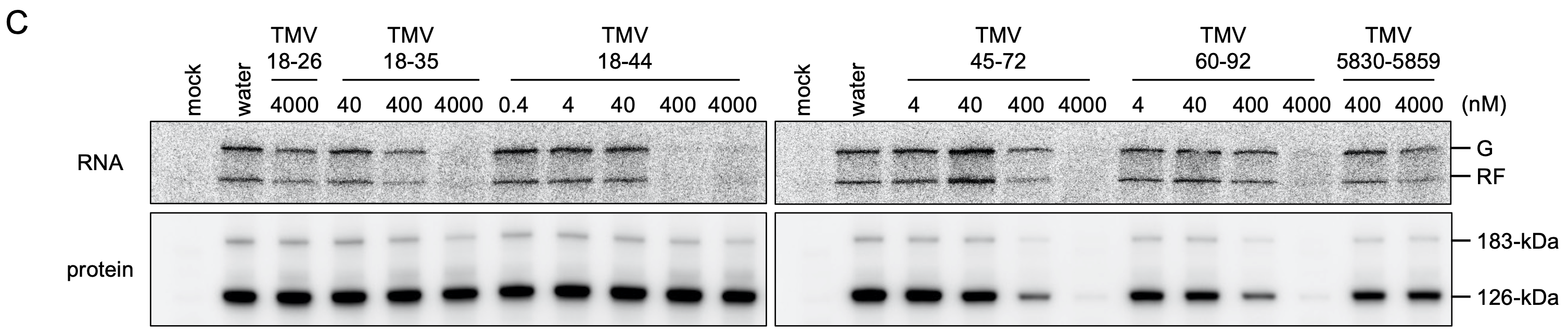

Supplement: Supplementary file 1 [file viruses-14-01962-s001.zip › FigS1-3/FigS2C.tiff]

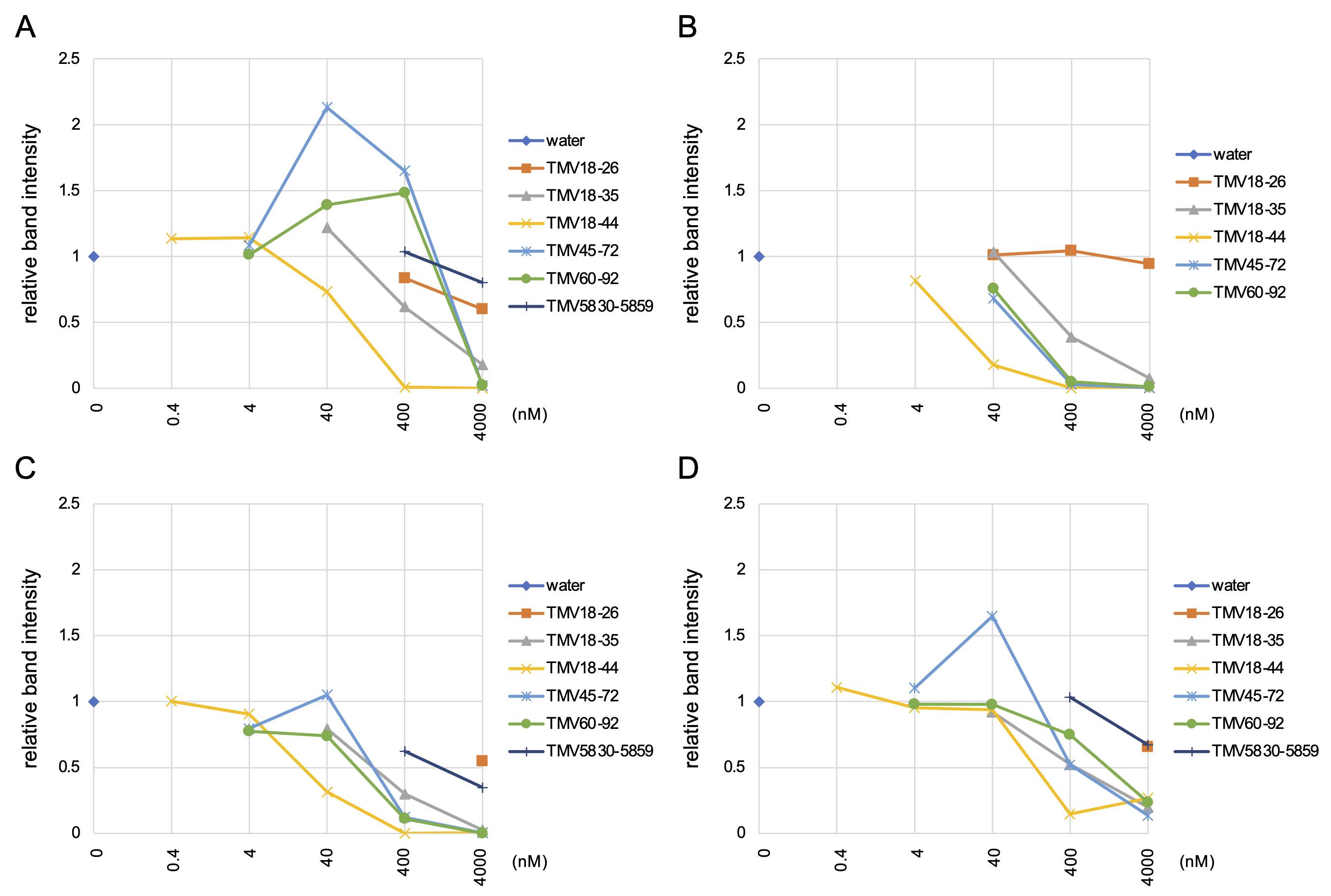

Supplement: Supplementary file 1 [file viruses-14-01962-s001.zip › FigS1-3/FigS3.tiff]
